# Physical functioning and mental health treatment initiation and retention for veterans with posttraumatic stress disorder: a prospective cohort study

**DOI:** 10.1186/s12913-021-07035-6

**Published:** 2021-09-23

**Authors:** Wei Duan-Porter, David B. Nelson, Kristine E. Ensrud, Michele R. Spoont

**Affiliations:** 1 Center for Care Delivery & Outcomes Research, Minneapolis VA Health Care System, One Veterans Dr, MN 55417 Minneapolis, United States; 2 University of Minnesota Medical School, MN Minneapolis, USA; 3 School of Public Health, University of Minnesota, MN Minneapolis, USA; 4 National Center for PTSD, Department of Veterans Affairs, Minneapolis, USA

**Keywords:** Physical limitations, Role limitations, Psychotherapy, PTSD symptoms, Multimorbidity

## Abstract

**Background:**

Most US adults with posttraumatic stress disorder (PTSD) do not initiate mental health treatment within a year of diagnosis. Increasing treatment uptake can improve health and quality of life for those with PTSD. Individuals with PTSD are more likely to report poor physical functioning, which may contribute to difficulty with treatment initiation and retention. We sought to determine the effects of poor physical functioning on mental health treatment initiation and retention for individuals with PTSD.

**Methods:**

We used data for a national cohort of veterans in VA care; diagnosed with PTSD in June 2008-July 2009; with no mental health treatment in the prior year; and who responded to baseline surveys on physical functioning and PTSD symptoms (n = 6,765). Physical functioning was assessed using Veterans RAND 12-item Short Form Health Survey, and encoded as limitations in physical functioning and role limitations due to physical health. Treatment initiation (within 6 months of diagnosis) was determined using VA data and categorized as none (reference), only medications, only psychotherapy, or both. Treatment retention was defined as having ≥ 4 months of appropriate antidepressant or ≥ 8 psychotherapy encounters.

**Results:**

In multinomial models, greater limitations in physical functioning were associated with lower odds of initiating only psychotherapy (OR 0.82 [95 % CI 0.68, 0.97] for limited a little and OR 0.74 [0.61, 0.90] for limited a lot, compared to reference “Not limited at all”). However, it was not associated with initiation of medications alone (OR 1.04 [0.85, 1.28] for limited a little and OR 1.07 [0.86, 1.34] for limited a lot) or combined with psychotherapy (OR 1.03 [0.85, 1.25] for limited a little and OR 0.95 [0.78, 1.17] for limited a lot). Greater limitations in physical functioning were also associated with lower odds of psychotherapy retention (OR 0.69 [0.53, 0.89] for limited a lot) but not for medications (e.g., OR 0.96 [0.79, 1.17] for limited a lot). Role limitations was only associated with initiation of both medications and psychotherapy, but there was no effect gradient (OR 1.38 [1.03, 1.86] for limitations a little or some of the time, and OR 1.18 [0.63, 1.06] for most or all of the time, compared to reference “None of the time”). Accounting for chronic physical health conditions did not attenuate associations between limitations in physical functioning (or role limitations) and PTSD treatment; having more chronic conditions was associated with lower odds of both initiation and retention for all treatments (e.g., for 2 + conditions OR 0.53 [0.41, 0.67] for initiation of psychotherapy).

**Conclusions:**

Greater limitations in physical functioning may be a barrier to psychotherapy initiation and retention. Future interventions addressing physical functioning may enhance uptake of psychotherapy.

## Introduction

Posttraumatic stress disorder (PTSD) affects more than 14 million US adults [1], but most of these individuals do not initiate treatment during the first year. Less than 10 % of those with PTSD obtain treatment within the first year of diagnosis, and for those who eventually seek treatment, the average time from symptom onset to treatment initiation is 4.5 years [1, 2]. Adults with PTSD have higher prevalence of chronic medical conditions, increased risk for adverse health outcomes, and greater difficulty with relationships at home and at work [3, 4]. Veterans experience PTSD at greater rates than the general population [3], and improving outcomes for veterans with PTSD has been a top priority for the Veterans Health Administration (VHA). Evidence-based treatments, including psychotherapy and appropriate medications, have been shown to substantially reduce PTSD symptoms in randomized trials with veterans and civilians [5–7]. While veterans with PTSD who are enrolled in VHA do engage in mental health treatment at a higher rate than non-veterans, a third still do not initiate psychotherapy or appropriate medications within 6 months of initial diagnosis [8]. Furthermore, only one-third or less (10–33 %) actually complete a sufficient trial of PTSD treatment [8, 9]. Some characteristics associated with differences in PTSD treatment initiation and retention include age, sex, health-related beliefs, and social support for treatment [8–10]. However, although individuals with PTSD are more likely to report poor physical functioning [11–13], it is unknown how physical functioning affects the likelihood of receiving PTSD treatments.

Physical functioning, an essential component of overall functioning, is generally lower for those with more chronic physical health conditions [14, 15]. Adults with PTSD are also more likely to report poor physical functioning, including limitations in mobility (e.g., climbing stairs) and difficulty completing common household chores [11–13]. Additionally, having more severe PTSD symptoms is associated with worse self-reported physical functioning [16]. It may be that PTSD symptoms, such as difficulty with sleep, directly contribute to fatigue and poor functioning. Also, certain coping strategies may lead to poor health behaviors [17, 18] that then negatively impact physical health and functioning over years. Thus, poor functioning may be due to both ongoing mental health symptoms and higher burden of chronic conditions among individuals with PTSD.

Difficulties with physical functioning may act as a barrier to attending clinic appointments, leading to more problems engaging in treatments that require frequent visits, such as psychotherapy. However, it is also possible that those with poor physical functioning are more likely to seek care for PTSD because they are more motivated to improve their overall health. Additionally, if poor physical functioning among those with PTSD is largely due to more chronic physical health conditions, then those with worse functioning may have more health care encounters and thus, opportunities to receive information and referrals for mental health care. Therefore, understanding the effect of physical functioning on PTSD treatment initiation and retention may support future efforts to enhance treatment uptake.

Using survey and VHA data for a prospective national cohort of veterans with PTSD, we examined whether self-reported physical functioning was associated with PTSD treatment initiation and retention. We evaluated effects of baseline physical functioning on initiation of PTSD treatment within 6 months of diagnosis, and whether effects were attenuated by adjustment for chronic physical health conditions. To evaluate impacts on treatment retention, we separately examined associations between physical functioning and having a minimally adequate trial of medications (among individuals who initiated medications), and of psychotherapy (among those who initiated therapy). We also determined whether controlling for chronic physical health conditions altered these associations.

## Methods

### Dataset & Participants

Detailed description of this national cohort of veterans with PTSD has been published previously [10]. Briefly, participants were veterans enrolled in VHA care with a diagnosis of PTSD (ICD-9 308.81) during an outpatient VHA encounter in June 2008-July 2009 with no psychiatric medications or mental health encounters in the prior year. Those with diagnoses of schizophrenia spectrum disorders or neurocognitive disorders were excluded. Women and racial-ethnic minorities were over-sampled for inclusion in the cohort. The primary objectives of this cohort study were to examine the association of health-related beliefs with receipt of PTSD treatments and changes in PTSD symptoms. There were 7,645 participants who completed the baseline survey, corresponding to a response rate of 66 % [10].

In these secondary analyses, we used baseline survey and VHA encounter and pharmacy data over 6 months after baseline to determine the association between physical functioning and likelihood of PTSD treatment initiation and retention. We excluded 869 participants who had missing responses to baseline survey items on physical functioning and/or PTSD symptoms. Compared with included participants, those who were excluded due to missing data for physical functioning and PTSD symptoms were overall older (mean age 59.1 years, SD 14.9, p < 0.001 by t-test), more often men (91 %, p < 0.001 by χ^2^-test), black (22 %, p < 0.001 by χ^2^-test), and had more chronic physical health conditions (22 % with one condition, 14 % with 2 or more, p < 0.001 by χ^2^-test). We also excluded 11 who died before 6 months follow-up, giving a final analytical sample of 6,765 individuals.

 This work was conducted in accordance with the Declaration of Helsinki and approved by the Minneapolis VA Health Care System Institutional Review Board. Informed consent was obtained from all participants at the time of baseline surveys.

### Measures

#### Outcomes—PTSD Treatment Initiation and Retention

We used VHA pharmacy and outpatient encounter data to determine receipt of PTSD treatments. For treatment initiation within 6 months, we encoded whether participants did not have any treatment (reference), received only medications (any recommended antidepressant—citalopram, escitalopram, sertraline, paroxetine, fluoxetine, duloxetine, venlafaxine, desvenlafaxine, or mirtazapine) [5], received only psychotherapy (had at least 1 outpatient encounter with CPT codes for individual or group therapy) [10], or had both medications and psychotherapy.

For treatment retention, we evaluated whether participants received a minimally adequate trial of at least 4 months of antidepressants or at least 8 individual and/or group psychotherapy sessions. These criteria are in agreement with current PTSD treatment guidelines [5] and have been applied in multiple past studies [8, 19].

#### Main Predictors—Physical Functioning

Baseline self-reported physical functioning was assessed using the Veterans 12-item RAND Short Form Health Survey (VR-12), which was adapted from the general population survey and has been validated for veterans [20, 21]. The VR-12 is derived from the 36-item Short Form survey (SF-36) and has conceptually distinct domains within physical functioning, including limitations in physical functioning and role limitations due to physical health [14].

For limitations in physical functioning, we encoded 3 categories using responses to 2 items on whether participants reported limitations in moderate activities (e.g., moving a table or using a vacuum cleaner) or climbing several flights of stairs. Response options were 3—“Limited a lot,” 2—“Limited a little,” and 1—“Not limited at all.” The reference category was no limitations reported for both questions. If the participant responded “Limited a lot” for either item, they were categorized as having the highest level of limitations. Those responding “Limited a little” to one or both items were included in a middle category denoting a relatively moderate level of limitations.

 For role limitations due to physical health, we similarly used 2 separate items asking how often participants accomplished less than they liked or limited the type of activities they did. Response options were on a 5-point Likert scale from 5—“All of the time” to 1—“None of the time.” We collapsed these responses into 3 categories, with the reference category being “None of the time” to both questions. If a participant reported one of the 2 highest response levels (“All of the time” or “Most of the time”) for either item, we coded their responses as the highest level of role limitations. All other response combinations were categorized as a more moderate level of role limitations.

#### Covariates

Participant demographics extracted from VHA administrative data included age, sex, race, and ethnicity. Baseline PTSD symptoms were measured by the PTSD Checklist–Military version (PCL-M), which is scored as the sum of responses to 17 items (score range 17–85) [22]. If participants had missing responses to individual items for PTSD symptoms but had data for at least 8 items, we imputed the summary score using the mean of available responses. Chronic physical health conditions were assessed using ICD-9 codes associated with VHA encounters in the year prior to baseline. Conditions included hypertension, diabetes mellitus, chronic lung disease, stroke, coronary artery disease, heart failure, valvular heart disease, peripheral vascular disease, cancer, neurologic conditions, renal failure, chronic liver disease, hypothyroidism, and anemia. Because each condition was relatively uncommon, we used the total number of conditions (categorized as 0, 1, or 2+) as a measure of overall burden of physical health conditions. These categories reflect established use of counts of conditions (both simple and weighted) as measures of comorbidity and in particular, multimorbidity (i.e., having 2 or more chronic conditions); higher counts are associated with greater health care needs and more treatment burden for patients [23, 24].

### Statistical Analyses

We first evaluated bivariate correlations between baseline demographic variables, physical functioning predictors (limitations in physical functioning and role limitations), PTSD symptoms (PCL-M), and chronic physical health conditions.

We then used multinomial logistic regression models to evaluate associations between baseline physical functioning and PTSD treatment initiation within 6 months. Separately for each physical functioning predictor, we fit a base model regressing treatment initiation on physical functioning, participant demographics (age, sex, race, and ethnicity), and baseline PTSD symptoms. Next, to evaluate whether associations were due to the relationship between multimorbidity and poor physical functioning, we added chronic physical health conditions (as 0, 1, or 2+) to the model. For all multinomial models, we present odds ratios (OR) and 95 % confidence interval (CI) for each treatment option (medications only, psychotherapy only, or both medications and psychotherapy) relative to having no treatment within 6 months. We also evaluated for interactions between baseline PTSD symptoms and physical functioning in predicting treatment initiation. Significant interactions were found for limitations in physical functioning and initiation of psychotherapy only; subsequently, we conducted additional stratified analyses assessing associations between physical functioning and treatment initiation by tertiles of PTSD symptom severity. No significant interactions were identified for role limitations and treatment initiation.

To evaluate the role of physical functioning on PTSD treatment retention, we used separate logistic models for having minimally adequate medications or psychotherapy among those who initiated each therapy. We fit basic and adjusted models analogous to those described above for treatment initiation. We similarly present OR and 95 % CI for all models predicting treatment retention.

All analyses were conducted in R version 4.0.2.

## Results

Baseline characteristics for participants are presented in Table 1. Most participants (80–93 %) reported at least some limitations in physical functioning or role limitations due to physical health. Mean age of participants was 50.1 years and 19 % had only one chronic physical health condition, while 13 % had 2 or more conditions. The most prevalent conditions were hypertension (22 %), diabetes mellitus (9 %), chronic lung disease (5 %), and coronary artery disease (5 %). On average, participants had moderate to severe PTSD symptoms at baseline (mean PCL-M 57.5, standard deviation [SD] 15.0) and poor mental health functioning (mean SF-12 Mental Component Score 33.4, SD 11.8).

**Table 1:** Baseline Characteristics of Participants (*N* = 6765)

Characteristics	Mean or *N* (% or SD)
*Demographics*	
Age	50.1 (16.2)
Sex^a^	
Women	1122 (17)
Men	5643 (83)
Race^a^	
White	2779 (41)
African American	1065 (16)
Native American or Native Alaskan	193 (3)
Asian American	183 (3)
Native Hawaiian or Pacific Islander	275 (4)
2+	112 (2)
Unknown	2158 (32)
Ethnicity^a^	
Hispanic or Latino	1348 (20)
Not Hispanic or Latino	3917 (59)
Unknown	1500 (22)
Marital Status	
Married or living with partner	4227 (62)
Not married, living alone	2479 (36)
Education	
< High school	277 (4)
High school graduate or GED	1643 (24)
Some college	3142 (46)
College graduate or higher	1647 (24)
Household Income	
<$10,000	1076 (16)
$10,000-$20,000	1398 (21)
$20,000-$40,000	2136 (32)
>$40,000	1898 (28)
*Physical Functioning*	
Limitations in physical functioning	
Not at all	1334 (20)
Limited a little	2861 (42)
Limited a lot	2580 (38)
Role limitations due to physical health	
None of the time	512 (8)
A little/some of the time	2752 (41)
Most/all of the time	3511 (52)
SF-12 Physical Component Score^b^	36.7 (11.3)
*Physical Health Conditions*	
Hypertension	1499 (22)
Diabetes mellitus	634 (9)
Chronic lung disease	306 (5)
Coronary artery disease	310 (5)
Number of chronic conditions:	
0	4579 (68)
1	1283 (19)
2+	903 (13)
*Mental Functioning and Symptoms*	
PTSD Checklist (PCL-M)^c^	57.5 (15.0)
SF-12 Mental Component Score^b^	33.4 (11.8)

*GED* General Educational Development certificate; *PCL-M* PTSD Checklist Military version; *PTSD* Posttraumatic Stress Disorder; *SD* standard deviation; *SF-12* Veterans Short Form 12-item Survey

^a^ From VA administrative data

^b^ Range 0-100, standardized score with general population mean = 50

^c^ Range 17–85

The baseline physical functioning predictors were correlated with each other (r = 0.71, p < 0.01); they were also moderately correlated with PCL-M (r = 0.34–0.42, p < 0.05) and age (r = 0.19–0.30, p < 0.05). Age and sex were correlated (r = 0.48, *p* < 0.05), with women participants being generally younger.

Overall, about half of participants (56 %, *N* = 3759) initiated at least one type of PTSD treatment within 6 months of follow-up: 15 % (n = 1037) initiated only medication, 19 % (*N* = 1305) had only psychotherapy, and 21 % (*N* = 1417) received both. Most participants (76 %-83 %) who initiated any treatment did so within 2 months after the baseline survey. Treatment retention was generally low. Among those who initiated medications, only 49 % (*N* = 1199 of 2454) met criteria for a minimally adequate trial (4 months or more). For those who initiated psychotherapy, even fewer had a sufficient trial—only 21 % (*N* = 576 of 2722) had at least 8 sessions. A summary of treatment initiation and retention by different baseline levels of physical functioning is provided in Table 2.

**Table 2:** Baseline Physical Functioning and PTSD Treatments Received

	Limitations in Physical Functioning^a^,N(%)		
	**Not at all** **(*N*= 1334)**	**Limited a little (*N*= 2858)**	**Limited a lot (*N*= 2573)**
Treatment Initiation:			
None	610 (45)	1279 (45)	1117 (43)
Medications only	179 (13)	433 (15)	425 (17)
Psychotherapy only	306 (23)	545 (19)	454 (18)
Both medications and psychotherapy	239 (18)	601 (21)	577 (22)
Medication Treatment Retention^b^	214 (16)	484 (17)	501 (19)
Psychotherapy Retention^c^	115 (9)	245 (9)	216 (8)
	**Role Limitations Due to Physical Health**^a^,N(%)		
	**None of** **the time** **(*N*= 512)**	**A little or Some of the time (*N*= 2750)**	**Most or All of the time (*N*= 3503)**
Treatment Initiation:			
None	271 (53)	1246 (45)	1489 (43)
Medications only	57 (11)	396 (14)	584 (17)
Psychotherapy only	114 (22)	561 (20)	630 (18)
Both medications and psychotherapy	70 (14)	547 (20)	800 (23)
Medication Treatment Retention^b^	62 (12)	434 (16)	703 (20)
Psychotherapy Retention^c^	30 (6)	232 (8)	314 (9)

^a^ Assessed using 2 items from the Veterans Short Form 12-item Survey; see Methods.

^b^ Retention defined as having at least 4 months of appropriate antidepressant medications.

^c^ Retention defined as having at least 8 psychotherapy visits, individual and/or group.

### Physical Functioning and PTSD Treatment Initiation

In initial multinomial models, greater limitations in physical functioning were associated with lower odds of initiating only psychotherapy (OR 0.82 [95 % CI 0.68–0.97] for limited a little, and OR 0.74 [95 % CI 0.61–0.90] for limited a lot; Table 3 A, Model 1). Limitations in physical functioning were not associated with odds of initiating only medications, or both medications and psychotherapy. Additional adjustment for chronic physical health conditions did not attenuate the associations between greater limitations and lower odds of psychotherapy initiation (Table 3 A, Model 2). In contrast, having a moderate level of role limitations was associated with higher odds of initiating both medications and psychotherapy (1.38 [95 % CI 1.03–1.86]; Table 3B, Model 1); adjustment for chronic conditions also did not alter this association (Table 3B, Model 2). In all models, more severe PTSD symptoms was associated with greater odds of treatment initiation (medications only, psychotherapy only, or both), and a higher number of chronic physical health conditions was associated with lower odds of treatment initiation (Table 3).

We also found significant interactions between PTSD symptom severity and limitations in physical functioning for initiation of only psychotherapy (p < 0.05). Therefore, we conducted analyses stratified by tertiles of baseline PTSD symptoms. The effect of limitations in physical functioning on odds of initiating only psychotherapy was most pronounced among those participants with more severe PTSD symptoms. For example, among those in the highest tertile of baseline PTSD symptoms, being limited a lot was associated with a 50 % reduction in the odds of initiating only psychotherapy (OR 0.52 [95 % CI 0.34–0.80], Table 4).

**Table 3:** Physical Functioning and PTSD Treatment Initiation within 6 Months.a

*A. Limitations in Physical Functioning*						
	**Model 1, OR (95 % CI)**			**Model 2, OR (95 % CI)**		
	**Medication Only**	**Therapy Only**	**Both**	**Medication Only**	**Therapy Only**	**Both**
PTSD symptoms (PCL-M)	**1.04** **(1.03, 1.04)**	**1.02** **(1.02, 1.03)**	**1.06** **(1.05, 1.06)**	**1.04** **(1.03, 1.04)**	**1.02** **(1.02, 1.03)**	**1.06** **(1.05, 1.06)**
Physical functioning^b^:						
Limited a little	1.04 (0.85, 1.28)	**0.82** **(0.68, 0.97)**	1.03 (0.85, 1.25)	1.06 (0.77, 1.47)	0.84 (0.70, 1.00)	1.06 (0.87, 1.29)
Limited a lot	1.07 (0.86, 1.34)	**0.74** **(0.61, 0.90)**	0.95 (0.78, 1.17)	1.14 (0.91, 1.43)	**0.81** **(0.66, 0.99)**	1.03 (0.83, 1.27)
# Chronic physical health conditions^c^:						
1	—	—	—	**0.58** **(0.48, 0.71)**	**0.43** **(0.36, 0.52)**	**0.49** **(0.41, 0.59)**
2+	—	—	—	**0.53** **(0.41, 0.67)**	**0.37** **(0.29, 0.46)**	**0.41** **(0.33, 0.52)**
*B. Role Limitations Due to Physical Health*						
	**Model 1, OR (95 % CI)**			**Model 2, OR (95 % CI)**		
	**Medication Only**	**Therapy Only**	**Both**	**Medication Only**	**Therapy Only**	**Both**
PTSD symptoms (PCL-M)	**1.03** **(1.03, 1.04)**	**1.02** **(1.02, 1.03)**	**1.06** **(1.05, 1.06)**	**1.03** **(1.03, 1.04)**	**1.02** **(1.02, 1.03)**	**1.06** **(1.05, 1.06)**
Role limitations^d^:						
A little or some of the time	1.31 (0.96, 1.80)	0.99 (0.78, 1.28)	**1.38** **(1.03, 1.86)**	1.33 (0.97, 1.82)	1.01 (0.79, 1.30)	**1.40** **(1.04, 1.89)**
Most or all of the time	1.29 (0.94, 1.79)	0.82 (0.63, 1.06)	1.18 (0.87, 1.60)	1.34 (0.97, 1.85)	0.86 (0.66, 1.12)	1.23 (0.90, 1.66)
# Chronic physical health conditions^c^:						
1	—	—	—	**0.58** **(0.48, 0.71)**	**0.43** **(0.36, 0.52)**	**0.49** **(0.41, 0.59)**
2+	—	—	—	**0.53** **(0.42, 0.67)**	**0.37** **(0.29, 0.46)**	**0.41** **(0.33, 0.52)**

*CI* confidence interval; *OR* odds ratio; *PCL-M* PTSD Checklist Military version; *PTSD* Posttraumatic Stress Disorder

^a^ All multinomial models evaluated the odds of initiating specific treatments (medications, psychotherapy, or both), with the reference as not initiating any treatment with 6 months. All models also included age, sex, race and ethnicity covariates, as determined by VA administrative data. OR in bold had p < 0.05

^b^ Reference is No limitations.

^c^ Reference is 0 (none).

^d^ Reference is None of the time.

**Table 4:** Limitations in Physical Functioning and PTSD Treatment Initiation within 6 Months—Models Stratified by Baseline PTSD Symptoms.a

	Odds Ratio (95 % CI)		
	**Meds Only**	**Therapy Only**	**Both**
*1st Tertile of PTSD Symptoms (PCL-M 17-52.2)*			
Physical functioning^b^:			
Limited a little	1.26 (0.91, 1.75)	1.08 (0.84, 1.40)	1.22 (0.89, 1.67)
Limited a lot	1.46 (0.99, 2.14)	1.07 (0.78, 1.46)	0.99 (0.66, 1.48)
*2nd Tertile of PTSD Symptoms (PCL-M 53–65)*			
Physical functioning^b^:			
Limited a little	1.00 (0.71, 1.42)	**0.69** **(0.51, 0.94)**	0.97 (0.70, 1.33)
Limited a lot	1.16 (0.80, 1.68)	0.73 (0.52, 1.01)	1.14 (0.81, 1.60)
*3rd Tertile of PTSD Symptoms (PCL-M 65.7–85)*			
Physical functioning^b^:			
Limited a little	0.83 (0.52, 1.32)	**0.60** **(0.39, 0.92)**	0.95 (0.63, 1.44)
Limited a lot	0.84 (0.54, 1.32)	**0.52** **(0.34, 0.80)**	0.90 (0.60, 1.34)

*CI* confidence interval; *OR* odds ratio; *PCL-M* PTSD Checklist Military version; *PTSD* Posttraumatic Stress Disorder

^a^ All multinomial models evaluated the odds of initiating specific treatments (medications, psychotherapy, or both), with the reference as not initiating any treatment with 6 months. All models also included age, sex, race and ethnicity covariates, as determined by VA administrative data. OR in bold had *p* < 0.05

^b^ Reference is No limitations.

### Physical Functioning and PTSD Treatment Retention

In initial models examining treatment retention, being limited a lot in physical functioning was associated with lower odds of retention in psychotherapy (OR 0.69 [95 % CI 0.53–0.89]); this effect was not attenuated by adjustment for chronic physical health conditions (Table 5). Limitations in physical functioning were not associated with medication retention, and role limitations were not associated with either medication or psychotherapy retention. In all models, more severe PTSD symptoms at baseline was associated with higher odds of treatment retention (Table 5).

**Table 5:** Physical Functioning and PTSD Treatment Retention

*A. Limitations in Physical Functioning*				
	**Medications,aOR (95 % CI)**		**Psychotherapyb,OR (95 % CI)**	
	**Model 1**	**Model 2**	**Model 1**	**Model 2**
PTSD symptoms (PCL-M)	**1.04** **(1.03, 1.04)**	**1.04** **(1.03, 1.04)**	**1.03** **(1.02, 1.03)**	**1.03** **(1.02, 1.03)**
Physical functioning^c^:				
Limited a little	0.94 (0.78, 1.13)	0.95 (0.79, 1.15)	0.85 (0.67, 1.08)	0.87 (0.68, 1.10)
Limited a lot	0.96 (0.79, 1.17)	1.01 (0.83, 1.22)	**0.69** **(0.53, 0.89)**	**0.73** **(0.56, 0.95)**
# Chronic physical health conditions^d^:				
1	—	**0.70** **(0.58, 0.84)**	—	**0.51** **(0.39, 0.66)**
2+	—	**0.62** **(0.49, 0.78)**	—	**0.49** **(0.35, 0.68)**
*B. Role Limitations Due to Physical Health*				
	Medications*, OR (95 % CI)		Psychotherapy*, OR (95 % CI)	
	**Model 1**	**Model 2**	**Model 1**	**Model 2**
PTSD symptoms (PCL-M)	**1.03** **(1.03, 1.04)**	**1.03** **(1.03, 1.04)**	**1.03** **(1.02, 1.03)**	**1.03** **(1.02, 1.03)**
Role limitations^e^:				
A little or some of the time	1.16 (0.87, 1.56)	1.17 (0.87, 1.57)	1.26 (0.85, 1.88)	1.28 (0.86, 1.90)
Most or all of the time	1.22 (0.91, 1.64)	1.24 (0.92, 1.67)	1.07 (0.72, 1.61)	1.11 (0.74, 1.66)
# Chronic physical health conditions^d^:				
1	—	**0.70** **(0.58, 0.84)**	—	**0.50** **(0.39, 0.66)**
2+	—	**0.62** **(0.49, 0.78)**	—	**0.48** **(0.35, 0.66)**

*CI* confidence interval; *OR* odds ratio; *PCL-M* PTSD Checklist Military version; *PTSD* Posttraumatic Stress Disorder

^a^ Logistic models were used to evaluate odds of having at least 4 months of appropriate antidepressant medications, among those who received any appropriate antidepressant (N = 2454). In addition to variables notes in table, all models also included age, sex, race and ethnicity covariates, as determined by VA administrative data. OR in bold had p < 0.05.

^b^ Logistic models were used to evaluate odds of completing at least 8 psychotherapy visits, among those who had at least 1 psychotherapy visit (group or individual; N = 2722). All models included the same set of additional variables as noted for medication models. OR in bold had *p* < 0.05.

^c^ Reference is No limitations.

^d^ Reference is 0 (none).

^e^ Reference is None of the time.

## Discussion

In this longitudinal study of veterans with PTSD, greater limitations in physical functioning were associated with decreased odds of psychotherapy initiation and retention. In contrast, limitations in physical functioning and role limitations were not associated with initiation or retention in medication treatment. Physical functioning may be a barrier for psychotherapy because the frequent session attendance required may be more burdensome for those who have difficulty with mobility and daily tasks. Additionally, we found evidence for interaction effects between baseline PTSD symptoms and limitations in physical functioning; the impact of physical functioning on psychotherapy initiation was greatest for individuals with the most severe symptoms. This suggests that limitations in physical functioning may be a more substantial barrier to psychotherapy for those who are the most affected by PTSD, and thus, have the greatest need for treatment. Furthermore, because individuals with PTSD often prefer psychotherapy over medications [5, 25, 26], poor physical functioning may be an important factor in whether many individuals will receive any treatment at all. Overall, our results indicate that poor physical functioning has independent (and detrimental) effects on psychotherapy initiation and retention, after accounting for effects of PTSD symptom severity.

Accounting for chronic physical health conditions did not substantially change associations between physical functioning and odds of psychotherapy initiation or retention. Thus, physical health comorbidities did not explain the relationships between physical functioning and psychotherapy initiation and retention. Moreover, having more physical health comorbidities was consistently associated with decreased odds for both medication and psychotherapy initiation and retention, suggesting that greater burden of chronic medical conditions is an independent barrier to PTSD treatment. Thus, in contrast to our expectation that having more conditions would result in more health care visits and potential opportunities for mental health referral, our results may indicate that the ensuing burden of managing multiple treatments and competing priorities may be particularly challenging for those with PTSD.

To our knowledge, this is the first study to evaluate the effects of physical health and functioning on likelihood of PTSD treatment initiation and retention. One prior study of the general US population showed that worse physical functioning was associated with greater likelihood of using any mental health services within one year; however, this cohort had a low prevalence of mental health symptoms and very low treatment utilization overall [27]. As expected, our cohort of veterans with PTSD had higher rates of mental health services utilization, although 44 % did not initiate any treatment within 6 months. Our results indicate that for those who are diagnosed with PTSD, it is important to account for the complex contributions of mental health symptoms, co-existing burden of chronic physical health conditions, and self-reported physical functioning on likelihood of receiving PTSD treatment.

Worse physical functioning and greater physical health burden may also worsen mental health symptoms, while acting as barriers to PTSD treatment uptake. The relationships between physical functioning and health on one hand, and psychological distress and mental health on the other, are likely complex. Physical health conditions can directly reduce capacity to complete daily tasks and maintain social roles, leading to additional experiences of emotional distress [28]. For those with PTSD, ongoing mental health symptoms related to traumatic exposures, poor physical functioning, and burden of physical health comorbidities may all contribute to a broader sense of helplessness and generalized distress.

Therefore, our results regarding the complex relationships between these factors support the need for greater integration of mental and physical health services. Enhanced longitudinal collaboration between mental health and primary care providers may be required to evaluate reasons for poor functioning and to develop effective treatment plans. VHA has taken important steps in this direction by implementing a co-located collaborative model of mental health services within primary care (Primary Care Mental Health Integration [PCMHI]) [29, 30]. However, while there have been some promising results showing increased initiation of PTSD treatments with PCMHI [29, 30], it is unknown if PCMHI has led to improvements in treatment retention or better outcomes.

Future work is also needed to better understand relationships between physical functioning, patient preferences for medications vs. psychotherapy, and provider recommendations for treatment. Furthermore, it may be important to consider the utility of psychotherapy via telehealth (i.e., sessions by telephone or video-conferencing), which may reduce barriers to attendance for those with PTSD and poor physical functioning. Although there have been evaluations of efficacy and acceptability for psychotherapy delivered via telehealth, there has been no evidence indicating improved adherence or uptake, particularly as compared with traditional in-person sessions [31–33]. During the COVID-19 pandemic, there have been rapid shifts to telehealth for a variety of psychotherapies in clinical settings [34]. Examination of patient outcomes and experiences with this natural experiment may also help deepen our understanding of the impacts associated with different delivery formats.

There are several limitations to our study. Although we adjusted for a range of conceptually important variables (e.g., age and chronic health conditions) that may act as confounders in understanding relationships between physical functioning and PTSD treatment uptake, we cannot rule out that our results were due to unobserved confounders. We used VHA encounter and pharmacy data to determine diagnoses of PTSD and receipt of mental health treatments. Thus, we do not know if participants adhered to treatments outside of attendance at psychotherapy sessions or dispensed medications (e.g., completed homework assignments or took prescribed medications). We also do not know if participants had non-VA treatments. However, VHA has considerable expertise in treating PTSD and is perceived as thus by veterans [35, 36]. Additionally, the majority of post-9/11 veterans who have accessed mental health treatments used VHA services [37]. Our study used data collected in 2009–2010, and as noted above, there have been ongoing efforts to improve VHA mental health services since that time. Beginning in 2008, VHA mandated implementation of PCMHI in VA primary care clinics [29], and in 2006–2007, launched national initiatives focused on training more mental health providers to deliver trauma-focused psychotherapies [38]. While PCMHI and these training initiatives were all in progress during the period of our study, the impact on uptake of PTSD treatments was probably not fully evident. However, it is likely that patient factors, including the role of poor physical functioning, are still relevant as barriers to treatment initiation and retention, even with increased VHA capacity to deliver mental health services. Our results may also not generalize to the general US population or to veterans not enrolled in VHA care; these individuals may encounter other barriers to treatment initiation and retention.

## Conclusions

We found that limitations in physical functioning may be a barrier to psychotherapy initiation and retention. Integrated care models may be better suited to address the complex needs of individuals with PTSD. Future work should address whether such models of care, or other interventions directly addressing physical functioning, will improve treatment uptake and outcomes for those with PTSD.

## Data Availability

The datasets used in the current study are not publicly available due to data security and privacy requirements for VHA data. They are available from the corresponding author on reasonable request.

## Abbreviations

CI: Confidence interval
OR: Odds ratio
PCL-M: PTSD Checklist–Military version
PTSD: Posttraumatic stress disorder
SD: Standard deviation
SF-36: 36 item Short-form Survey
VA: Department of Veterans Affairs
VHA: Veterans Health Administration
VR-12: Veterans 12-item RAND Short Form Health Survey

## References

[1] Goldstein RB, Smith SM, Chou SP, Saha TD, Jung J, Zhang H, Pickering RP, Ruan WJ, Huang B, Grant BF (2016). The epidemiology of DSM-5 posttraumatic stress disorder in the United States: results from the National Epidemiologic Survey on Alcohol and Related Conditions-III. Soc Psychiatry Psychiatr Epidemiol.

[2] Wang PS, Berglund P, Olfson M, Pincus HA, Wells KB, Kessler RC (2005). Failure and delay in initial treatment contact after first onset of mental disorders in the National Comorbidity Survey Replication. Arch Gen Psychiatry.

[3] 2014 Institute of Medicine (2014). Treatment for Posttraumatic Stress Disorder in Military and Veteran Populations: Final Assessment.

[4] Pacella ML, Hruska B, Delahanty DL (2013). The physical health consequences of PTSD and PTSD symptoms: a meta-analytic review. J Anxiety Disord.

[5] The Management of Post-Traumatic Stress Working Group: VA/DoD Clinical Practice Guideline for Management of Post-Traumatic Stress Disorder and Acute Stress Disorder. In.; 2017.

[6] Taylor S, Thordarson DS, Maxfield L, Fedoroff IC, Lovell K, Ogrodniczuk J (2003). Comparative efficacy, speed, and adverse effects of three PTSD treatments: exposure therapy, EMDR, and relaxation training. J Consult Clin Psychol.

[7] Schnurr PP, Friedman MJ, Engel CC, Foa EB, Shea MT, Chow BK, Resick PA, Thurston V, Orsillo SM, Haug R (2007). Cognitive behavioral therapy for posttraumatic stress disorder in women: a randomized controlled trial. JAMA.

[8] Spoont MR, Murdoch M, Hodges J, Nugent S (2010). Treatment receipt by veterans after a PTSD diagnosis in PTSD, mental health, or general medical clinics. Psychiatr Serv.

[9] Seal KH, Maguen S, Cohen B, Gima KS, Metzler TJ, Ren L, Bertenthal D, Marmar CR (2010). VA mental health services utilization in Iraq and Afghanistan veterans in the first year of receiving new mental health diagnoses. J Trauma Stress.

[10] Spoont MR, Nelson DB, Murdoch M, Rector T, Sayer NA, Nugent S, Westermeyer J (2014). Impact of treatment beliefs and social network encouragement on initiation of care by VA service users with PTSD. Psychiatr Serv.

[11] Zatzick DF, Marmar CR, Weiss DS, Browner WS, Metzler TJ, Golding JM, Stewart A, Schlenger WE, Wells KB (1997). Posttraumatic stress disorder and functioning and quality of life outcomes in a nationally representative sample of male Vietnam veterans. Am J Psychiatry.

[12] Magruder KM, Frueh BC, Knapp RG, Johnson MR, Vaughan JA, Carson TC, Powell DA, Hebert R (2004). PTSD symptoms, demographic characteristics, and functional status among veterans treated in VA primary care clinics. Journal of Traumatic Stress.

[13] Byers AL, Covinsky KE, Neylan TC, Yaffe K (2014). Chronicity of posttraumatic stress disorder and risk of disability in older persons. JAMA Psychiatry.

[14] Ware JE, Sherbourne CD (1992). The MOS 36-item short-form health survey (SF-36). I. Conceptual framework and item selection. Med Care.

[15] Hays RD, Marshall GN, Wang EY, Sherbourne CD (1994). Four-year cross-lagged associations between physical and mental health in the Medical Outcomes Study. J Consult Clin Psychol.

[16] Schnurr PP, Hayes AF, Lunney CA, McFall M, Uddo M (2006). Longitudinal analysis of the relationship between symptoms and quality of life in veterans treated for posttraumatic stress disorder. J Consult Clin Psychol.

[17] Zen AL, Whooley MA, Zhao S, Cohen BE (2012). Post-traumatic stress disorder is associated with poor health behaviors: findings from the heart and soul study. Health Psychol.

[18] van den Berk-Clark C, Secrest S, Walls J, Hallberg E, Lustman PJ, Schneider FD, Scherrer JF (2018). Association between posttraumatic stress disorder and lack of exercise, poor diet, obesity, and co-occuring smoking: A systematic review and meta-analysis. Health Psychol.

[19] Wang PS, Lane M, Olfson M, Pincus HA, Wells KB, Kessler RC (2005). Twelve-month use of mental health services in the United States: results from the National Comorbidity Survey Replication. Arch Gen Psychiatry.

[20] Kazis LE, Miller DR, Clark J, Skinner K, Lee A, Rogers W, Spiro A, Payne S, Fincke G, Selim A (1998). Health-related quality of life in patients served by the Department of Veterans Affairs: results from the Veterans Health Study. Arch Intern Med.

[21] Kazis LE, Miller DR, Skinner KM, Lee A, Ren XS, Clark JA, Rogers WH, Sprio A, Selim A, Linzer M (2006). Applications of methodologies of the Veterans Health Study in the VA healthcare system: conclusions and summary. J Ambul Care Manage.

[22] Weathers F, Litz B, Herman D, Keane T: PTSD Checklist: Military Version (PCL-M). In.: National Center for PTSD; 1994.

[23] Johnston MC, Crilly M, Black C, Prescott GJ, Mercer SW (2019). Defining and measuring multimorbidity: a systematic review of systematic reviews. Eur J Public Health.

[24] Boyd CM, Darer J, Boult C, Fried LP, Boult L, Wu AW (2005). Clinical practice guidelines and quality of care for older patients with multiple comorbid diseases: implications for pay for performance. Jama.

[25] Roy-Byrne P, Berliner L, Russo J, Zatzick D, Pitman RK (2003). Treatment preferences and determinants in victims of sexual and physical assault. J Nerv Ment Dis.

[26] Zoellner LA, Feeny NC, Bittinger JN (2009). What you believe is what you want: modeling PTSD-related treatment preferences for sertraline or prolonged exposure. J Behav Ther Exp Psychiatry.

[27] Ware JE, Manning WG, Duan N, Wells KB, Newhouse JP (1984). Health status and the use of outpatient mental health services. Am Psychol.

[28] Stanners MN, Barton CA, Shakib S, Winefield HR: Depression diagnosis and treatment amongst multimorbid patients: a thematic BMC Fam Pract 2014, 15:124. 10.1186/1471-2296-15-124 PMC4074384 24947875

[29] Wray LO, Szymanski BR, Kearney LK, McCarthy JF (2012). Implementation of primary care-mental health integration services in the Veterans Health Administration: program activity and associations with engagement in specialty mental health services. J Clin Psychol Med Settings.

[30] Bohnert KM, Sripada RK, Mach J, McCarthy JF (2016). Same-Day Integrated Mental Health Care and PTSD Diagnosis and Treatment Among VHA Primary Care Patients With Positive PTSD Screens. Psychiatr Serv.

[31] Morland LA, Wells SY, Glassman LH, Greene CJ, Hoffman JE, Rosen CS: Advances in PTSD Treatment Delivery: Review of Findings and Clinical Considerations for the Use of Telehealth Interventions for PTSD. Curr Treat Options Psychiatry 2020:1–21. 10.1007/s40501-020-00215-x PMC7261035 32837831

[32] Olden M, Shingleton R, Finkelstein-Fox L, Peskin M, Cukor J, Ovalles A, Rabinowitz T, Difede J (2016). Telemedicine Exposure Therapy and Assessment for PTSD: a Systematic Clinical Practice Narrative Review. Journal of Technology in Behavioral Science.

[33] Thomas N, McDonald C, de Boer K, Brand RM, Nedeljkovic M, Seabrook L (2021). Review of the current empirical literature on using videoconferencing to deliver individual psychotherapies to adults with mental health problems. Psychol Psychother.

[34] Li H, Glecia A, Kent-Wilkinson A, Leidl D, Kleib M, Risling T: Transition of Mental Health Service Delivery to Telepsychiatry in Response to COVID-19: A Literature Review. Psychiatr Q 2021. 10.1007/s11126-021-09926-7 PMC8185490 34101075

[35] Rosenheck R, Fontana A (1995). Do Vietnam-era veterans who suffer from posttraumatic stress disorder avoid VA mental health services?. Mil Med.

[36] Sayer NA, Friedemann-Sanchez G, Spoont M, Murdoch M, Parker LE, Chiros C, Rosenheck R (2009). A qualitative study of determinants of PTSD treatment initiation in veterans. Psychiatry.

[37] Elbogen EB, Wagner HR, Johnson SC, Kinneer P, Kang H, Vasterling JJ, Timko C, Beckham JC: Are Iraq and Afghanistan veterans using mental health services? New data from a national random-sample survey. Psychiatr 2013, 64(2):134–141. 10.1176/appi.ps.004792011 PMC3622866 23475498

[38] Karlin BE, Ruzek JI, Chard KM, Eftekhari A, Monson CM, Hembree EA, Resick PA, Foa EB (2010). Dissemination of evidence-based psychological treatments for posttraumatic stress disorder in the Veterans Health Administration. J Trauma Stress.

